# Effects of Laser Annealing Parameters on Optical and Electrical Properties of ITO/Metallic Glass Alloy Bi-layer Films

**DOI:** 10.1186/s11671-015-0982-4

**Published:** 2015-06-30

**Authors:** H. K. Lin, K. C. Cheng, J. C. Huang

**Affiliations:** Graduate Institute of Materials Engineering, National Pingtung University of Science and Technology, 1, Hseuhfu Road, Pingtung, 912 Taiwan; Department of Materials and Optoelectronic Science, National Sun Yat-Sen University, 70, Lien-Hai Road, Kaohsiung, 804 Taiwan

**Keywords:** Laser, Transparent conductive film, Metallic glass, Optical property, Electric resistivity

## Abstract

AgAlMg (AAM) films with three different atomic percentage compositions are prepared, namely, Ag_12_Al_62_Mg_26_ (denoted as A1AM), Ag_22_Al_46_Mg_32_ (denoted as A2AM), and Ag_36_Al_25_Mg_39_ (denoted as A3AM). In addition, the AAM films are deposited with four different thicknesses, i.e., 3, 6, 9, and 12 nm. The indium-tin oxide thickness is assigned a constant value of 30 nm in every case. The results show that the optical transmittance of the AAM/IAAM films improves (i.e., increases) with a reducing AAM film thickness, while the electrical resistivity improves (i.e., reduces) with an increasing film thickness. It is shown that the IA2AM film with an AMM thickness of 9 nm yields the optimal compromise between the optical transmittance and the electrical resistivity. The as-deposited IAAM films are found to have optical transmittance and electric resistivity values of 65 % and 90 Ω/□, respectively. The IA2AM films are annealed using a near-infrared laser at different pulse energies with a wavelength of 1064 nm and repetition rates ranging from 100 ~ 400 kHz. For both films, the optical and electrical properties are enhanced as the pulse energy increases to a certain critical value due to a transition from an amorphous microstructure to a crystalline structure. Given a repetition rate of 400 kHz and a pulse energy of 1.03 μJ, the optical transmittance and sheet resistance of the IAAM film are found to be 80 % and 15 Ω/□, respectively. The corresponding value of the Haacke figure of merit changed from 0.15 × 10^−3^ to 7.16 × 10^−3^ Ω^−1^ due to the optimal laser annealing conditions.

## Background

Transparent conducting oxide (TCO) films have a low electrical resistivity and a high optical transmittance in the visible range and are therefore widely used for such electronic applications as flat panel displays, organic light-emitting diodes (OLEDs), cholesteric liquid crystal displays, touch panels, and solar cells. Because of the various TCO films available, indium-tin oxide (ITO) is one of the most commonly applied for optoelectronic devices due to its excellent electrical and optical properties and its ease of preparation using physical vapor deposition techniques [1–5]. However, to minimize the sheet resistance, the thickness of the ITO film should exceed 100 nm [6, 7]. Consequently, the use of pure ITO films for optoelectronic devices is cost prohibitive. To address this problem, the literature contains many proposals for minimizing the ITO cost by means of ITO-metal layer-ITO sandwich structures. Typically, these structures are based on the use of highly conductive metals such as silver or copper as the middle layer. In general, the results have shown that a metal layer thickness of around 5 ~ 20 nm yields both good optical transmittance in the visible light range and a high conductivity [8–13].

Bulk metallic glasses (BMGs) possess an amorphous structure and have many advantageous properties, including high strength, excellent hardness, and anticorrosion. Therefore, BMGs have an extensive range of potential applications in the industrial, electronics, and biomedicine fields. For example, Inoue [14] examined the mechanical, chemical, and magnetic properties of BMGs produced using various casting processes. In addition, three empirical rules for BMGs were proposed, namely (1) multi-component structures consisting of at least three elements, (2) atomic size mismatches of at least 12 % among the three main elements, and (3) negative heats of mixing among the three main elements. Various thin film metallic glasses (TFMGs) have been developed in recent years. These TFMGs exhibit a lower surface roughness than pure metallic films [15] and have many unique properties, including high strength, mechanical properties, and physical properties [16–18]. Notably, metallic glass films have high nucleation rate and a thickness much lower than that of the metal layers used in traditional ITO sandwich structures. Consequently, the use of TFMGs to realize thin transparent conductor films with excellent transparency and conductivity properties has attracted growing interest in recent years.

It is well known that the optical and electrical properties of thin films can be improved through annealing [19–21]. As a result, the literature contains various proposals for the localized annealing of TCO films using pulsed laser systems [22–26]. For example, Lin and Hsu [26] patterned ITO thin-film electrodes using a high-repetition-rate fiber laser and showed that the residual stress reduced as the repetition rate increased.

Lee et al. [27] deposited an ITO/ZrCu bi-layer film on a PET substrate using a magnetron sputtering technique. It was shown that a continuous and smooth ZrCu layer with a thickness of less than 6 nm could be obtained given an appropriate choice of sputtering conditions. Moreover, the ZrCu film had an optical transmittance of 73 % for incident light with a wavelength of 550 nm. However, ZrCu metallic glass films have a relatively high sheet resistance. Accordingly, in the present study, the ZrCu film is replaced with a metallic glass alloy film comprising three low-resistivity components, namely, silver (Ag), aluminum (Al), and magnesium (Mg). The study considers both AgAlMg (AAM) monolithic films and ITO/AgAlMg (IAAM) bi-layer films. The investigation focuses particularly on the effects of the AAM composition and AAM film thickness on the optical transmittance and electric resistivity of the monolithic and bi-layer structures. The as-deposited AAM and IAAM films are then annealed using a fiber laser with pulse repetition rates ranging from 100 to ~400 kHz. The optimal laser annealing parameters are determined by evaluating the electrical, optical, and structural properties of the various samples using four-point probe technique, spectrophotometry, scanning electron microscopy (SEM), and X-ray diffraction (XRD).

## Methods

The AAM and IAAM films were deposited on glass substrates using a magnetron sputtering system (Kao Duen Co.). The glass substrates were purchased from Nippon Electric Glass Co. and had a thickness of 0.7 mm and an optical transmittance of 90 % for an incident wavelength of 550 nm. The IAAM films were deposited by means of a co-sputtering technique using an ITO target with a composition of 90 wt.% In_2_O_3_ and 10 wt.% SnO_2_, and pure Ag, Al, and Mg targets, respectively. All of the targets had a diameter of 2 in.. The sputtering process was performed using an Ar flow rate of 30 sccm, a base pressure of 2 × 10^−6^ Torr, and a working pressure of 5 mTorr. In synthesizing the IAAM films, AgAlMg films with a thickness of 3 ~ 12 nm were deposited on the glass substrate, and an ITO layer with a thickness of 30 nm was then sputtered on the metallics glass (MG) layer. To evaluate the effects of the composition of the MG layer on the optical and electrical properties of the AAM and IAAM films, the discharge power of the Ag target was varied in the range of 25 to 37 W in order to obtain three different AAM films, namely, Ag_12_Al_62_Mg_26_ (denoted as A1AM), Ag_22_Al_46_Mg_32_ (denoted as A2AM), and Ag_36_Al_25_Mg_39_ (denoted as A3AM). Note that the composition is specified in terms of the atomic percentage in every case.

The optimal optical and resistance of the AAM and IAAM films were annealed using a fiber laser (SPI-12, UK) with a wavelength of 1064 nm and repetition rates ranging from 100 to ~400 kHz. Moreover, the irradiation power was set in the range of 42 ~ 545 mW and the scanning speed was set as 30 and 50 mm/s in order to anneal at the similar overlapping rate. The laser system had a spot size of 42 μm, a pulse duration of 30 ns, and a scanning pitch of 25 μm. For each annealing process, the pulse energy (*E*) was computed as [28]

1 $$ E={P}_{\mathrm{AVG}}/\mathrm{rep}, $$

where *P*_AVG_ and rep denote the average power of the pulse laser and the laser repetition rate, respectively. From Eq. (1), it is seen that the pulse energy reduces as the repetition rate increases. For the irradiation powers and repetition rates considered in the present study, the pulse energy varied from 0.4 to 1.4 μJ. Moreover, the overlapping rate used to control the distribution of the individual laser pulses on the thin film surface was computed as

2 $$ \mathrm{Overlapping}=\left(D-S\right)/D\times 100\%, $$

where *D* is the laser spot diameter, and *S* is the length of two successive laser spots and is dependent on the repetition rate and the scanning speed. For the laser spot diameter, repetition rates, and scanning speeds used in the present annealing tests, the overlapping rate was determined to be around 99 %. The morphologies and compositions of the as-deposited and annealed AAM and IAAM films were examined by SEM (JSM-7600F) and in-plane XRD (Bruker D8 Advance). The sheet resistance was measured using a four-point probe (SR-H1000C). Finally, the optical transmittance was measured over the range of 200 ~ 1100 nm using a UV-vis-IR spectrophotometer (Lambda 35, PerkinElmer).

## Results and Discussion

**Fig. 1:**
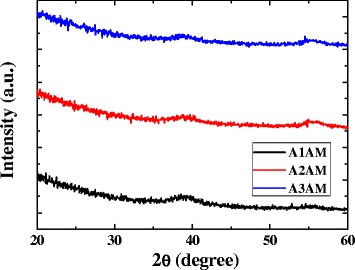
X-ray diffraction scans of AAM monolithic films

**Fig. 2:**
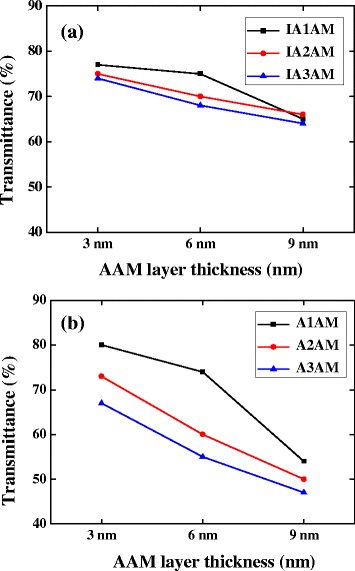
Optical transmittance of **a** IAAM films and **b** AMM films as a function of AAM film thickness

Figure 1 shows the X-ray diffraction patterns for the A1AM, A2AM, and A3AM films. The absence of a distinct diffraction peak in any of the XRD profiles indicates that all three AAM films have an amorphous structure. Figure 2a, b shows the variation of the transmittance with the AAM film thickness for the IAAM bi-layer films and AAM monolithic films, respectively. The results confirm that for both films, the transmittance reduces with an increasing MG thickness due to the corresponding increase in the reflectivity of the MG layer. In addition, it is seen that with the exception of the IA1AM film with a thickness of 9 nm, the transmittance of the IAAM films and AAM films reduces with an increasing Ag content. Her and Wang [29] reported that the optical transmittance of Al films is higher than that of Ag films of the same thickness. For the present AAM films, the Al content decreases as the Ag content increases. Thus, as the Ag content is increased from 12 to ~ 36 at.%, the transmittance decreases. It is noted that this tendency is consistent with that reported in [29] for Ag films on glass substrates.

**Fig. 3:**
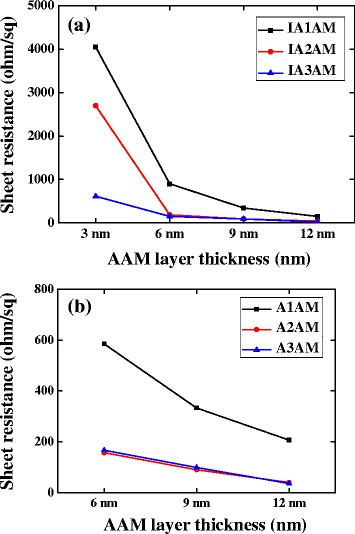
Sheet resistance of **a** IAAM films and **b** AMM films as a function of AAM film thickness

Figure 3 shows the sheet resistance values of the IAAM and AAM films as a function of the AAM film thickness and Ag content. For both films, the sheet resistance decreases with an increasing AAM thickness due to a greater continuity of the film structure. Moreover, the resistance also reduces with an increasing Ag content. From inspection, the A3AM and IA3AM films with an AAM thickness of 12 nm have a sheet resistance of 36 and 18 Ω/□, respectively.

**Fig. 4:**
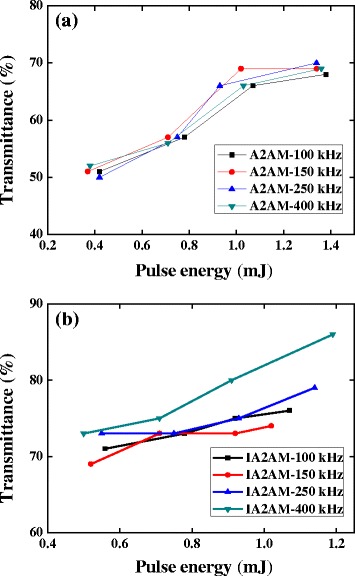
Optical transmittance of annealed samples on the glass substrate as function of pulse energy and repetition rate: **a** AAM films and **b** IAAM films

It is shown that the IAAM film with an AAM thickness of 9 nm yields the optimal compromise between the optical transmittance and the electrical resistivity. Therefore, the IA2AM film was then annealed at different laser conditions. Figure 4 shows the optical transmittance of the A2AM and IA2AM films following annealing with various pulse energies and repetition rates. Note that the incident wavelength is 550 nm in every case. It is seen that for both films, the transmittance increases with an increasing pulse energy. For example, the transmittance of the A2AM sample increases from around 50 to 70 % as the pulse energy is increased from 0.4 to 1.4 μJ. For the IA2AM sample, the transmittance is around 80 % given a repetition rate of 400 kHz and a pulse energy of 1.2 μJ. In other words, the annealing process improves the transmittance by around 23 % compared to the as-deposited sample.

**Fig. 5:**
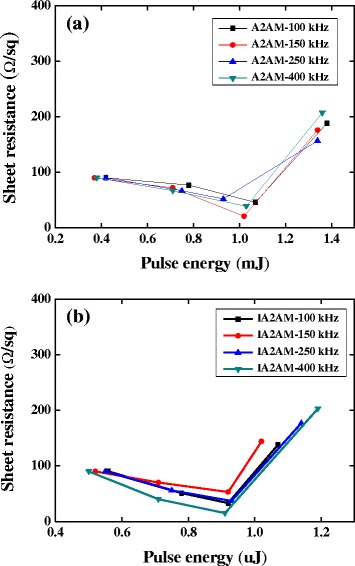
Sheet resistance of annealed samples on the glass substrate as function of pulse energy and repetition rate: **a** AAM films and **b** IAAM films

Figure 5 shows the sheet resistance of the A2AM and IA2AM samples following laser annealing with various pulse energies and repetition rates. Observing the two figures, it is seen that as-deposited films have a sheet resistance of approximately 90 Ω/□. Moreover, it is observed that the sheet resistance reduces as the pulse energy is increased toward a certain threshold value but then increases thereafter. For the AMM film, the minimum sheet resistance has a value of approximately 21 Ω/□ and is obtained using a pulse energy of 1 μJ and a repetition rate of 150 kHz. Similarly, for the IA2AM film, the minimum sheet resistance is equal to 15 Ω/□ and occurs using a pulse energy of 0.91 μJ and a repetition rate of 400 kHz. For both films, the sheet resistance increases significantly as the pulse energy is increased beyond the critical threshold value. As discussed below, this is the result of an ablation effect induced by the intense energy input, which results in the destruction of the film surface.

**Fig. 6:**
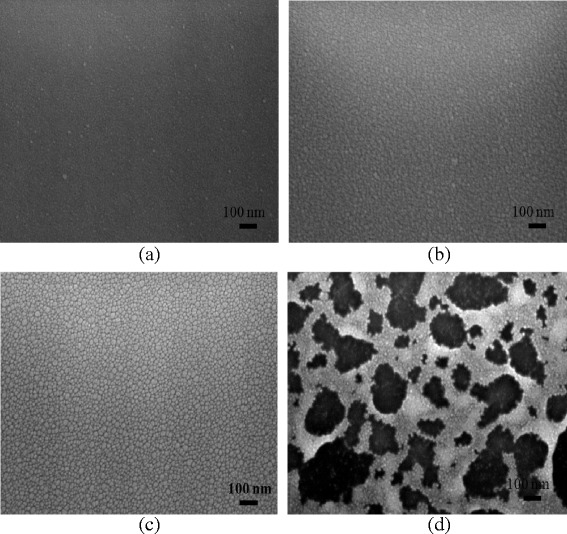
SEM images of A2AM films annealed with repetition rate of 150 kHz and pulse energies of **a** 0.37, **b** 0.71, **c** 1.02, and **d** 1.34 μJ

Figure 6 presents a series of SEM images showing the surface of the A2AM films annealed using a constant repetition rate of 150 kHz and pulse energies ranging from 0.37 to ~1.34 μJ. It is seen that for the lowest pulse energy of 0.37 μJ, the sample retains an amorphous structure. Thus, the electrical and optical properties of the A2AM film are similar to those of the as-deposited sample. As the pulse energy is increased to 1.02 μJ, the microstructure transforms from an amorphous structure to a crystalline structure with a fine grain size. Higuchi et al. [30] reported that the resistivity decreased with increasing domain size. The domain boundary might cause scattering for conduction electrons. Therefore, larger domain ITO films had a higher Hall mobility. Crystallographic orientation of the ITO films showed a clear relationship with refractive indices [31]. As the films were annealed, they become denser and have lower refractive indices. Granqvist et al. [32] also reported that the extinction coefficient accounts for the absorption in the longer wavelengths, the higher the extinction coefficient, and lower the transmittance. Consequently, both the electrical resistivity and the optical transmittance of the annealed film are improved. However, as the pulse energy is further increased to 1.34 μJ, the surface of the A2AM film is damaged by the excessive energy input. As a result, the electrical properties of the sample are seriously degraded.

**Fig. 7:**
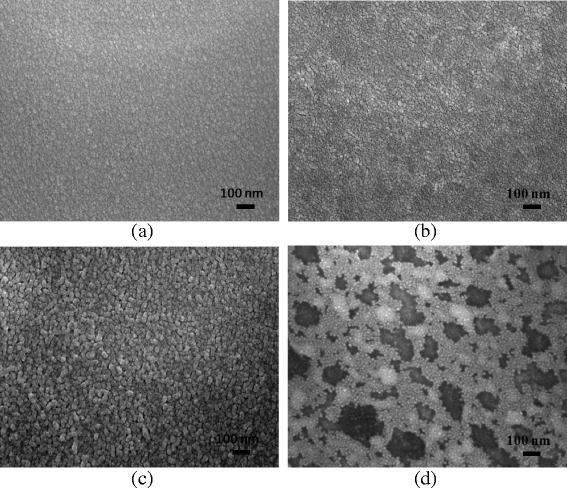
SEM images of IA2AM films annealed with repetition rate of 400 kHz and pulse energies of **a** 0.5, **b** 0.71, **c** 0.91, and **d** 1.19 μJ

Figure 7 presents SEM images showing the surface morphologies of the IA2AM samples annealed at a constant repetition rate of 400 kHz and pulse energies of 0.5 ~ 1.19 μJ. As with the A2AM film, the microstructure of the IAAM bi-layer film transforms to a fine crystalline structure as the pulse energy is increased to 0.91 μJ. Consequently, the sheet resistance reduces, while the optical transmittance increases. However, at a higher pulse energy of 1.19 μJ, the film surface contains many voids. As a result, the optical transmittance is improved, but the electrical conductivity is significantly reduced.

**Fig. 8:**
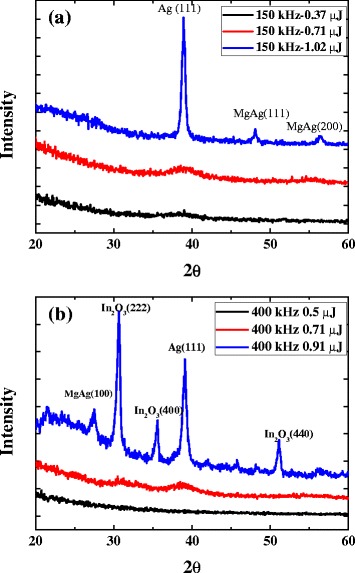
XRD profiles of samples annealed under different laser conditions: **a** A2AM films and **b** IA2AM films

Figure 8 presents the XRD analysis results for the annealed A2AM and IA2AM films. The XRD profiles in Fig. 8a confirm that the A2AM film retains an amorphous structure or ultra-fine grain size given a pulse energy of less than 0.71 μJ and a repetition rate of 150 kHz. However, for a higher pulse energy of 1.02 μJ, the XRD profile contains a sharp peak corresponding to Ag(111). In other words, the SEM observation of a crystalline A2AM structure given a repetition rate of 150 kHz and a pulse energy of 1.02 μJ is confirmed. The XRD profiles in Fig. 8b show that the IA2AM samples annealed with a repetition rate of 400 kHz retain an amorphous structure or ultra-fine grain size for pulse energies of less than 0.71 μJ. However, for a higher pulse energy of 0.91 μJ, the XRD profile contains broad peaks corresponding to the (100), (222), (400), and (111) orientations. In other words, the ITO/metallic glass bi-layer has a crystalline characteristic (see Fig. 7c).

Overall, the results presented in Figs. 2, 3, 4, and 5 show that the electrical and optical properties of the IAAM bi-layer film are superior to those of the AAM monolithic film. Moreover, the results suggest that the optimal tradeoff between the electrical performance and the optical performance of the IAAM film is obtained given a repetition rate of 400 kHz and a pulse energy of 0.91 μJ. Haacke [33] argued that the performance of transparent conductive films with known sheet resistance and transmittance properties could be compared via the following figure of merit:

3 $$ {\upphi}_{\mathrm{TC}}={T}^{10}/R, $$

where *T* is the transmittance (expressed in percentage terms) and *R* is the sheet resistance (expressed in units of Ω/□). Considering the application of the annealed IA2AM films to the display field, the transmittance at 550 nm light wavelength is used. Substituting the transmittance and sheet resistance values of the as-deposited IA2AM and annealed sample with a repetition rate of 400 kHz and a pulse energy of 0.91 μJ (i.e., 80 % and 15 Ω/□, respectively) into Eq. (3), the figure of merit is found to be 0.15 × 10^−3^ to 7.16 × 10^−3^ Ω^−1^, respectively. This high value of Ψ_TC_ confirms the optimality of the laser annealing conditions.

## Conclusions

Monolithic AAM metallic glass films and bi-layer ITO/AAM metallic glass films have been deposited on glass substrates using a magnetron sputtering process. The investigation has focused specifically on the effects of the Ag content (12 ~ 36 at.%) and AAM film thickness (3 ~ 12 nm) on the optical transmittance and sheet resistance of the synthesized samples. With increasing AAM film thickness from 3 to 12 nm, for both films, the optical transmittance increases with a reducing film thickness, whereas the electrical resistance reduces with an increasing film thickness. Moreover, for a constant film thickness, the optical transmittance and electrical resistance both reduce as the Ag content is increased. The results have shown that an IAAM film comprising an ITO layer with a thickness of 30 nm and an Ag_22_Al_46_Mg_32_ film with a thickness of 9 nm provides the optimal compromise between the optical and electrical properties of the film. The as-deposited films have been annealed using a pulsed fiber laser system with various repetition rates and pulse energies. The results have shown that for both films (AAM and IAAM), the amorphous structure of the as-deposited samples transforms to a crystalline structure as the pulse energy is increased. However, as the pulse energy is increased beyond a certain critical value, the excessive energy input causes significant damage of the film surface. It has been shown that the crystalline structure improves the electrical and optical properties of the annealed samples compared to those of the amorphous as-deposited films. For the A2AM film, the electrical resistivity and optical transmittance have optimal values of 21 Ω/□ and 70 % given a repetition rate of 150 kHz and a pulse energy of 1.02 μJ. Similarly, for the IA2AM film, the optimal values of the electrical resistivity and optical transmittance are 15 Ω/□ and 80 %, respectively, given a repetition rate of 400 kHz and a pulse energy of 0.91 μJ. For both films, the electrical resistivity increases significantly as the pulse energy is increased beyond the corresponding threshold value due to the damage caused to the film surface. Overall, the results presented in this study show that the addition of a 30-nm ITO film to a thin (9 nm) AAM metallic glass alloy structure yields a significant improvement in the electrical and optical properties of the thin-film structure. The corresponding value of the Haacke figure of merit changed from 0.15 × 10^−3^ to 7.16 × 10^−3^ Ω^−1^ due to the optimal laser annealing conditions. Thus, the present IAAM bi-layer structure represents an ideal solution for a wide variety of TCO applications.
